# LAPAROSCOPIC AND LAPAROTOMY BARIATRIC SURGERY IN A PUBLIC HOSPITAL IN BRAZIL: ARE THERE DIFFERENCES IN COSTS AND COMPLICATIONS?

**DOI:** 10.1590/0102-672020230021e1739

**Published:** 2023-06-02

**Authors:** Wagner Augusto Schiel, Antônio de Pádua Peppe, André Gubert Weiss, Luís Gustavo Guides Cortiano, Alcides José Branco, Francisco Emanuel Almeida, Mateus Rocco

**Affiliations:** 1 Santa Casa de Misericórdia Hospital, Department of Bariatric and Metabolic Surgery – Curitiba (PR), Brazil.

**Keywords:** Laparoscopy, Bariatric surgery, Laparotomy, Costs and cost analysis, Obesity, morbid, Postoperative complications, Laparoscopia, Cirurgia bariátrica, Laparotomia, Custos e análise de custo, Obesidade mórbida, Complicações pós-operatórias

## Abstract

**BACKGROUND:**

Despite its increasing popularity, laparoscopy is not the option for bariatric surgeries performed in the Brazilian public health system.

**AIMS:**

To compare laparotomy and laparoscopic access in bariatric surgery, considering aspects such as morbidity, mortality, costs, and length of stay.

**METHODS:**

The study included 80 patients who were randomly assigned to perform a Roux-en-Y gastric bypass. They were equally divided in two groups, laparoscopic and laparotomy. The results obtained in the postoperative period were evaluated and compared according to the Ministry of Health protocol, and later, in their outpatient returns.

**RESULTS:**

The surgical time was similar in both groups (p=0.240). The costs of laparoscopic surgery proved to be higher, mainly due to staplers and staples. The patients included in the laparotomy group presented higher rates of severe complications, such as incisional hernia (p<0.001). Costs related to social security and management of postoperative complications were higher in the open surgery group (R$ 1,876.00 vs R$ 34,268.91).

**CONCLUSIONS:**

The costs related to social security and treatment of complications were substantially lower in laparoscopic access when compared to laparotomy. However, considering the operative procedure itself, the laparotomy remained cheaper. Finally, the length of stay, the rate of complications, and return to labor had more favorable results in the laparoscopic route.

## RESUMO

**RACIONAL:**

Apesar de sua crescente popularidade, a laparoscopia não é a via de acesso em cirurgias bariátricas realizadas no sistema público de saúde brasileiro.

**OBJETIVOS:**

Comparar os acessos laparoscópico e laparotômico em cirurgia bariátrica, considerando aspectos como morbidade, mortalidade, custos e tempo de hospitalização.

**MÉTODOS:**

Foram incluídos 80 pacientes candidatos a by-pass gástrico em Y-de-Roux, aleatoriamente divididos em dois grupos, de acordo com a via de acesso. Os resultados obtidos no período pós-operatório foram avaliados e comparados de acordo com o protocolo do Ministério da Saúde, e posteriormente, em seus retornos ambulatoriais.

**RESULTADOS:**

O tempo cirúrgico foi semelhante em ambos os grupos (p=0.240). Os custos da cirurgia laparoscópica foram maiores, principalmente devido aos grampeadores e cargas. Contudo, os pacientes designados à via aberta apresentaram maior índice de complicações graves, como hérnia incisional (p<0.001). Desta forma, os custos com o tratamento das complicações e com o seguro social foram maiores neste grupo (R$ 1,876.00 vs R$ 34,268.91).

**CONCLUSÃO:**

Os gastos relacionados ao seguro social e ao tratamento de complicações foram substancialmente menores na cirurgia laparoscópica quando comparada à cirurgia aberta. Entretanto, considerando o procedimento operatório em si, a via aberta foi a mais acessível financeiramente. Por fim, o tempo de hospitalização, a taxa de complicações e o tempo de retorno ao trabalho tiveram resultados mais favoráveis na via laparoscópica.

## INTRODUCTION

Morbid obesity is an serious health problem in Brazil^4,5^. According to DATASUS, obesity (body mass index (BMI) >30 kg/m^2^) affects approximately 16% of the Brazilian population, which is more than twice the prevalence of 20 years ago^3^. Behavioral measures, such as diet and exercise, rarely lead to long-term weight loss when applied alone. Pharmacological treatments often fail and many lack proven safety. In addition, studies confirm the resolution of comorbidities associated with obesity after bariatric surgery^1,14^.

According to the 1991 North American National Institution of Health (NIH) Consensus Conference, surgical procedures are the only effective treatments for morbid obesity in patients with a BMI greater than 40 kg/m^2^ or in patients with a BMI greater than 35 kg/m^2^ associated with weight-related comorbidities. Since 1991, Roux-en-Y gastric bypass has become increasingly popular^6,14^.

The Roux-en-Y gastric bypass by laparotomy is the most performed procedure for the treatment of morbid obesity in the Brazilian Unified Health System (SUS)^8^. This procedure was first described in 1994 by Wittgrove et al.^13^ and has been undertaken more frequently due to the high demand of patients. Several studies were carried out aiming to report the mortality and complications of each procedure separately. Almost all of these studies were subject to publication controversies since most of the evidence is drawn from studies derived from individual institutions with small samples, or despite the large volume, they were restricted to a specific access route^15^.

Therefore, the limitations of administrative data set do not enable the comparison of laparoscopic versus laparotomy as shown in the publications, including the Medicare database and the United States Nationwide Admission Sample^2,12,16^.

Although both surgeons and patients currently show preferences for the laparoscopic technique, it is still not available for the SUS patients. There are no randomized, prospective, risk-adjusted studies comparing the results of laparoscopic and open gastric bypass surgery for morbid obesity in SUS patients. The lack of scientific evidence comparing both techniques in terms of efficacy and morbidity keeps laparoscopic access still out of coverage.

The objective of this study was to evaluate the feasibility of laparoscopic bariatric surgery compared to the laparotomy, considering morbidity, mortality, length of hospital stay, and hospitalization costs in the 30-day, 3-month, 6-month and 12-month periods.

## METHODS

This a randomized cohort study, approved by the Ethics Committee of the Pontifícia Universidade Católica do Paraná (number of 2.348.495). The period of study was 18 months.

Eighty obese patients with indication of bariatric surgery were included, according to the following criteria: age between 18 and 55 years; BMI >35 and <50 kg/m² (obesity grade II or III); preoperative endocrinological, cardiological, surgical, nutritional and psychological or psychiatric evaluation; patients with diabetes mellitus type 2, less than 10 (ten) years of the disease; no previous upper abdominal surgery; without other serious morbidities, which may offer an increase in the anesthetic risk related to the laparoscopic procedure; possibility of clinical follow-up for 12 months; and signature of the Term of Free and Informed Consent after properly informed about risks and possible complications of the procedure by the health team.

The exclusion criteria used were: surgical risk according to the American Society of Anesthesiologists (ASA) >2; pregnancy or lactation; any contraindication for the surgical treatment of obesity; presence of active infection; cancer in progression or any other chronic or acute progressive disease with a reserved prognosis.

The patients were equally and randomly assigned in laparoscopic and laparotomy groups. After the procedure, the patients were followed up and evaluated during their hospital stay and ambulatory returns, according to the Ministry of Health protocol for postoperative of bariatric surgery.

The order of the study was as follows: 1. Selection of morbidly obese patients according to inclusion criteria; 2. Application of a standardized questionnaire to participants; 3. Signature of informed consent; 4. Multi-professional consultation (endocrinologist, cardiologist, surgeon, and psychologist); 5. Performing preoperative exams; 6. Conduct of procedures; and 7. Postoperative follow-up for 12 months (return visits in 15- to 30-day, 2-month, 3-month, 4-month, 6-month, 9-month and 12-month periods). During the visits, routine clinical and laboratory parameters were evaluated, as well as endoscopic and imaging parameters, according to the specific indication and then, data analysis.

The project was developed together with the Advanced Center of Videolaparoscopy of Paraná (CEVIP) and the Bariatric and Metabolic Surgery Service of the Santa Casa de Misericórdia Hospital in Curitiba (PR). The procedures were undertaken at the Santa Casa de Misericórdia Hospital in Curitiba (PR), which had infrastructure and experience in operations of high complexity.

All the procedures were standardized between the two groups, differing only by the access route. After general anesthesia, intermittent pneumatic compression boots were installed for prophylaxis of deep venous thrombosis (DVT), as performed routinely in bariatric surgery.

The randomization procedure was applied by means of a simple draw of sealed envelopes, without external identification, at the moment preceding the beginning of the operation.

After the procedure, the length of hospital stay, analgesic use, total cost of hospitalization, visual pain scale, clinical complications (pulmonary or urinary infections, pulmonary atelectasis, venous thromboembolism) and surgical complications (digestive hemorrhage, fistula, abdominal wall) were evaluated and recorded.

In the first postoperative day, it was performed: fasting maintenance, intravenous analgesia with simple analgesics and opioids; general clinical evaluation; and early motor and respiratory physiotherapy.

As for the second postoperative day, the patients received: oral diet introduction, fractionally restricted liquid for both groups; general clinical evaluation; motor and respiratory physiotherapy; simple intravenous analgesics and opioids if needed; and hospital discharge at afternoon if good tolerance to pain and diet.

For the third postoperative day, patients took: an oral diet, restricted and fractional liquid for both groups; general clinical evaluation; motor and respiratory physiotherapy; simple intravenous analgesics and opioids if needed; and hospital discharge in the morning if good tolerance to pain and diet.

During outpatient follow-up, the patients were evaluated according to the routine established by the Ministry of Health protocol for postoperative bariatric surgery (Annex I), plus an additional evaluation at the end of the third postoperative month. All returns analyzed the occurrence of surgical and clinical complications, quality of life, and time of return to usual work activities.

The data collected in this study were compiled into Microsoft Excel® tables, using Epi Info™ software for statistical analysis. The quantitative variables were described as mean, median, minimum and maximum values, and qualitative variables by frequencies and percentages. For the comparison of the groups, concerning qualitative variables, the Fisher’s exact test and the Pearson’s chi-square (χ²) tests were considered. Regarding quantitative variables, the groups were compared using Student’s *t*-test for independent samples. The normality condition (p<0.05) was assessed by the Kolmogorov-Smirnov (K-S) test. The data were analyzed using the IBM Statistical Package for Social Sciences (SPSS) v.20.0 software.

## RESULTS

Of the 80 patients participating in the study, 40 were submitted to Roux-en-Y bypass through laparotomy (Group 1) and 40 by laparoscopic surgery (Group 2). The greatest number of participants were female (91.25%). The average age of Group 1 (G1) was 40.0 years, similar to Group 2 (G2), 39.5 years.

The average BMI, as well as the mean preoperative abdominal circumference (AC), were also similar between the two groups. G1 had a mean BMI of 40.9 and AC of 116.0 cm, while G2 had a mean BMI of 41.6 and AC of 119.5 cm. (Table 1).

**Table 1:** Measures variables.

	Surgery	n	Average	Median	Minimum	Maximum	Standard deviation	p-value
Age (years)	Open Video	40 40	40.0 39.5	39.5 39.0	21.0 22.0	62.0 60.0	9.8 11.2	0.857
Weight (kg)	Open Video	40 40	104.0 108.8	102.5 107.0	80.0 93.0	133.0 127.0	13.6 10.5	0.102
Height (m)	Open Video	40 40	1.6 1.6	1.6 1.6	1.5 1.5	1.7 1.8	0.1 0.1	0.114
Abdominal circumference (cm)	Open Video	40 40	116.0 119.5	115.5 121.0	96.0 109.0	142.0 127.0	11.7 4.9	0.119
Body mass index (kg/m)	Open Video	40 40	40.9 41.6	40.1 40.6	35.0 37.5	49.0 47.2	4.0 3.1	0.390

In G1 there were 18 patients with grade II obesity (45%) and 22 patients with grade III (55%). G2 had 6 patients classified as grade I obesity (15%), 16 as grade II (40%), and 18 as grade III (45%).

Statistical data on the comorbidities present in the preoperative period of both groups can be observed in Table 2.

**Table 2:** Preoperative comorbidities.

Comorbidities	Group 1	Group 2	p-value
	n (%)	n (%)	
Dyslipidemia	25 (62.5)	19 (47.5)	0.303
Glucose intolerance	17 (42.5)	10 (25.0)	0.155
Diabetes Mellitus – Type II	7 (17.5)	10 (25.0)	0.586
Esophagitis	18 (45.0)	20 (50.0)	0.331
Gastroesophageal reflux disease	15 (37.5)	21 (52.5)	0.280
Asthma	2 (5.0)	2 (5.0)	1.000
Hepatic steatosis	22 (56.4)	16 (56.3)	0.029
Non-hepatic liver disease	27 (67.5)	14 (35)	0.007
Cholelithiasis	6 (15.0)	6 (15.0)	1.000
Urinary incontinence	15 (37.5)	14 (35.0)	1.000
Polycystic ovary syndrome	0	4 (10)	0.116
Psychiatric disorder	2 (5.0)	10 (25.0)	0.025
Sleep apnea	0	4 (10.0)	0.016
Intertrigo	15 (37.5)	10 (25.0)	0.033
Smoking	9 (22.5)	4 (10.0)	0.231
Abdominal hernia	2 (5.0)	2 (5.0)	1.000
Hypertension	19 (47.5)	19 (47.5)	1.000

Patients with chronic renal failure (CRF), gastric and/or duodenal ulcer, intestinal polyps, HIV, endocrinological or pneumological restrictions were not observed in any of the groups. Only one patient from G1 did not obtain psychiatric and psychological release due to depressive disorder.

Regarding preoperative anesthetic consultation, in the G1, three patients were classified as ASA I (7.89%) and 35 as ASA II (92.11%). In G2, all patients were classified as ASA II (100%). About the cardiologic evaluation, in G1, 36 patients were classified as Goldman low (94.74%) and 2 as moderate (5.26%). In the G2, 26 patients were classified as Goldman low (86.67%) and 4 as moderate (13.33%).

There was a small difference in the surgical procedure time between the groups (85.1 min in G1 and 80.8 min in G2), however, without no significant statistical difference (p=0.240). The surgical technique used was the same, differing only in the access route and amount of stapler loads.

In both groups, the intestinal anastomosis segment section was performed with linear stapler with one load, and the entero-entero anastomosis was anisoperistaltic latero-lateral.

Different amounts of stapler loads were used to perform the gastric section. In G1, 39 patients used 2 loads, 1 patient used 1 load and no patient needed 4 loads. In G2, only 2 patients used 2 loads, 36 patients used 3 loads and 2 patients used 4 loads. (Table 3).

**Table 3:** Stapler loads used for stomach section.

Stomach loads	Laparotomy n (%)	Laparoscopy n (%)
2	39 (97.5)	2 (5.0)
3	1 (2.5)	36 (90)
4	0 (0.0)	2 (5.0)

Regarding transoperative complications, gastric suture bleeding was observed in 22.5% of patients in G1 and none in G2 (p=0.001). The results of postoperative complications are shown in Table 4.

**Table 4:** Postoperative complications.

Postoperative complications	Group 1 n (%)	Group 2 n (%)	p-value
Nausea	15 (37.5)	10 (25.0)	0.033
Vomiting	10 (25.0)	2 (5.0)	0.025
Wound bruise	0	8 (20.0)	0.005
Incisional hernia	7 (17.5)	0	<0.001
Shallow intermittent	7 (17.5)	1 (2.5)	<0.050
Gastro-jejunal stenosis	3 (7.5)	2 (5.0)	0.850

All patients in G1 received prophylaxis for deep venous thrombosis and pulmonary embolism with 40 mg of Enoxaparin once a day, in the morning. In G2, 42.5% received 40 mg and 57.5% received 60mg of the same drug, once a day. None of the patients received prophylaxis with Enoxaparin every 12 hours. Only one patient in G1 required rehospitalization due to thromboembolism, with good evolution.

Neither of the groups reported any occurrence of aponeurotic dehiscence, fistulas, intestinal obstructions, abscesses, or infectious, electrolytic, hemorrhagic, cardiac, and renal complications. No patients included in the study needed reoperation or died during the follow up.

Comparing the costs, the differences of the amounts spent between the procedures were basically the costs of the staplers. The stapler plus four loads cost about R$ 2,400.00 for the laparotomy (G1). For the laparoscopic procedure (G2), 8 loads plus the *LigaSure*™ (Maryland, Medtronic) cost about R$ 8,900.00. In G1, an average of 3.0 loads per surgery were used, compared with 4.0 loads in the G2. Therefore, the cost of surgical material in G2 was significantly higher.

Regarding the costs associated with postoperative complications, G1 required 3 endoscopic dilations due to gastro-jejunal stenosis, adding R$ 2,814.00 (R$ 938,00 per procedure). Besides stenosis, G1 increased the costs with the treatment of incisional hernias, because 7 patients were submitted to herniorrhaphies (R$ 539.92 per surgical procedure plus R$ 400.00 for each prolene mesh 15x15 cm x 7), adding R$ 6,579.44.

The only case of thromboembolism that required hospitalization was a patient of the G1, that raised the hospital costs in R$ 521.33. Therefore, the surgical complications of the patients of the G1 raised the hospital costs in R$ 9,914.77. In the G2, only 2 endoscopic dilations due to gastro-jejunal stenosis were required, raising the hospital costs in R$ 1,876.00.

In G1, the average time for the discharge was 2.86 postoperative day, generating a cost of R$ 2,350.00 with hospital services, while in G2 it was 2.0 days and the costs, R$ 1,643.33.

Comparing the time of absence from work activities, G1 remained on average 49.5 days apart, while G2 remained only 14.5 days. The social security costs supported by the SUS due to the time away from work presented a significant difference (p<0.05) between the groups; whereas G1 spent R$ 24,354.14, the G2 caused no expenses for the time away from activities.

The G1 had a substantially higher social security and complication costs totalizing R$ 34,268.91, while G2 summed R$ 1,876.00. Surgical material costs were significantly higher in G2, resulting in a difference of R$ 260,000.00 over G1.

About the follow-up of patients in 15-30 days, 2 months, 3 months, 4 months, 6 months, 9 months and 12 months, the result of BMI loss was very similar in both groups.

## DISCUSSION

Both groups were similar regarding gender, age, AC, BMI and number of patients, which offered greater credibility to the study. Surgical time was similar as well. Certainly, the increasing technical and technological evolution of video-surgery enabled procedures as agile as that of traditional laparotomy.

A major limitation of most studies on the subject is the morbimortality approach, but not the efficacy of laparoscopy versus open-label procedures. There is no information assessment on other important results metrics, as return to normal activity or work^3^.

The present study evidenced the superiority of the laparoscopic access over the laparotomy in terms of length of hospital stay and time away from work activities.

The laparoscopic Roux-en-Y, due to its minimally invasive nature, reduced postoperative hospitalization time by more than 30%. The Roux-en-Y laparotomy presented higher morbidity in the patients of our study, causing higher rates of complications, such as the occurrence of incisional hernia (17.5% in G1 vs 0% in G2), vomiting (25% in G1 vs 10% in G2), and superficial subcutaneous infection (17.5% in G1 vs 2.5% in G2).

Reoch et al.^9^, in a meta-analysis published in the Journal of the American Medical Association (JAMA), stated that laparoscopic bariatric surgery had significantly lower rates of incisional hernia and wound infection than open surgery, and the overall risk of mortality was similar between the two groups. However, this was a questionable result, due to the wide 95% confidence interval (95%CI 0.22–3.28)^9^.

On the other hand, incisional hernias may occur later at the trocar sites after laparoscopic bypass in obese patients. Rossi et al. analyzed 123 patients with BMI >35 kg/m^2^, submitted to Roux-en-Y gastric bypass laparoscopy and postoperative follow-up for at least 6 months. All patients were called up for an evaluation by the surgeon on the existence of incisional hernias in the trocar sites through physical examination and abdominal ultrasonography. A total of seven hernias were detected by physical examination, while ultrasonography detected a total of 56, in at least one of the incisions sites^10^.

Several studies indicated superiority of the laparoscopic route in relation to the open access regarding complications and length of hospital stay^9,15^. Prospective, multicenter data obtained from the Private Hospital Associations showed that laparoscopic gastric bypass is safer than open gastric bypass compared to 30-day complication rates^3^. In the present study we presented similar data on the rate of postoperative complications and shorter hospital stay in the group that underwent laparoscopic surgery.

Some other studies confirmed the cost-effectiveness of bariatric surgery^11^. Although the costs of the laparoscopic procedure are greater than that of open surgery^15^, the total value of hospital admission is lower^7^. We can observe data similar to the literature found in our study. The cost differences between each group, is basically in the cost with the staplers. While the open track used an average of 3.0 loads generating an average cost of R$ 2,400.00, the laparoscopic access used an average of 4.0 charges, with a cost of R$ 8,900.00. However, when considering the SUS social security, charges and cost of postoperative complications, the laparoscopic route presented a lower cost (R$ 1,876.00 in G2 vs R$ 34,268.91 in G1).

A possible limitation of this study would be the different proportion of comorbidities presented in both groups. In G1 there was a higher percentage of dyslipidemia. In G2, there was a higher prevalence of gastroesophageal reflux disease, osteoarthropathy, and stress urinary incontinence. The other diseases had similar prevalence between the groups.

## CONCLUSIONS

Based on the data presented, laparoscopic surgery was superior to the open route, considering the reduction of postoperative complications, early return to daily activities and work, as well as the reduction of social security by the SUS costs, hospitalization and patient care. However, the costs of laparoscopic procedure are still higher due to the staplers values. In addition, further studies are needed in this field, so that the patient’s well-being is always guaranteed and preferably with the best possible cost-benefit.

## References

[1] Buchwald H, Avidor Y, Braunwald E, Jensen MD, Pories W, Fahrbach K (2004). Bariatric surgery: a systematic review and meta-analysis. JAMA.

[2] Flum DR, Salem L, Elrod JA, Dellinger EP, Cheadle A, Chan L (2005). Early mortality among Medicare beneficiaries undergoing bariatric surgical procedures. JAMA.

[3] Goodman C. (1988). Medical technology assessment directory: a pilot reference to organizations, assessments, and information resources.

[4] Griz LH, Viégas M, Barros M, Griz AL, Freese E, Bandeira F (2010). Prevalence of central obesity in a large sample of adolescents from public schools in Recife, Brazil. Arq Bras Endocrinol Metabol.

[5] Hedley AA, Ogden CL, Johnson CL, Carroll MD, Curtin LR, Flegal KM (2004). Prevalence of overweight and obesity among US children, adolescents, and adults, 1999-2002. JAMA.

[6] Malafaia AB, Nassif PAN, Lucas RWDC, Garcia RF, Ribeiro JGA, Proença LB, Mattos ME, Ariede BL (2022). Is the waist/height ratio a better parameter than BMI in determining the cardiometabolic risk profile of obese people?. Arq Bras Cir Dig.

[7] Nguyen NT, Goldman C, Rosenquist CJ, Arango A, Cole CJ, Lee SJ (2001). Laparoscopic versus open gastric bypass: a randomized study of outcomes, quality of life, and costs. Ann Surg.

[8] Raabe FP (2014). Dissertação (Mestrado).

[9] Reoch J, Mottillo S, Shimony A, Filion KB, Christou NV, Joseph L (2011). Safety of laparoscopic vs open bariatric surgery: a systematic review and meta-analysis. Arch Surg.

[10] Rossi FMB, Moreno R, Druziani AL, Perez MM, Possari E, Ferreira da-Silva RB (2022). Incisional hernia after bariatric surgery: only the physical examination is enough?. Arq Bras Cir Dig.

[11] Salem L, Jensen CC, Flum DR (2005). Are bariatric surgical outcomes worth their cost? A systematic review. J Am Coll Surg.

[12] Santry HP, Gillen DL, Lauderdale DS (2005). Trends in bariatric surgical procedures. JAMA.

[13] Schirmer B (2006). Laparoscopic bariatric surgery. Surg Endosc.

[14] Smith BR, Schauer P, Nguyen NT (2011). Surgical approaches to the treatment of obesity: bariatric surgery. Med Clin North Am.

[15] Weller WE, Rosati C (2008). Comparing outcomes of laparoscopic versus open bariatric surgery. Ann Surg.

[16] Zingmond DS, McGory ML, Ko CY (2005). Hospitalization before and after gastric bypass surgery. JAMA.

